# Effect of nanogold incorporation into polymethyl methacrylate denture bases on microbial activity in implant-retained mandibular overdentures

**DOI:** 10.1186/s40729-024-00579-2

**Published:** 2025-01-06

**Authors:** Yasmin S. Zidan, Reham H. Abdel-Hamid, Reham M. Elshiekh, Sara M. El Gohary

**Affiliations:** Lecturer at removable prosthodontic department, Faculty of dental medicine for Girls, Al-Azhar University, Cairo, Egypt

**Keywords:** Implant, Nanogold, Antimicrobial activity

## Abstract

**Purpose:**

In this randomized clinical trial, we examined the incorporation of nanogold particles into polymethyl methacrylate denture bases and compared these modified bases with conventional ones in mandibular implant-retained overdentures, focusing on microbiological growth and adhesion characteristics.

**Methods:**

In this study, twenty-two male patients who were completely edentulous participated in a rehabilitation program involving mandibular overdentures retained by two dental implants placed in the canine area. The subjects were categorized into two equal groups, each comprising eleven patients. Group I received mandibular overdentures fabricated from conventional acrylic denture bases, whereas Group II received mandibular overdentures with bases that had undergone nanogold treatment. Microbial growth and colonization were evaluated around the implant’s necks and the fitting surface of each patient’s mandibular dentures. Three types of bacteria were studied: Candida albicans, Escherichia coli, and Streptococcus mutans. The mean difference in the counts of bacteria before the denture was inserted and after two, four, and six months has been calculated and analyzed statistically.

**Results:**

Regarding colony count (log 10 CFUs/mmL), there was a significant difference between the research groups. Group II had significantly lower values measured at 2, 4, and 6 months for Candida albicans, Escherichia coli, and Streptococcus mutans, respectively, than group I.

**Conclusion:**

The addition of gold nanoparticles to PMMA denture bases was of greater benefit in inhibiting microbial growth than conventional acrylic resin bases.

## Background

Complete edentulism represents the final stage of a complex interplay of various biological and patient-specific factors. Losing the teeth can have a number of adverse impacts on an individual’s overall social and psychological satisfaction, including a reduction in life quality, damage to the mouth’s structure, and disruption of oral functions [1].

Even with effective implementation, oral rehabilitating with a conventional complete denture fails to entirely solve the functional and psychological problems that completely edentulous patients face. The most common complaints are poor retention, insufficient support, and instability of the mandibular denture, which leads to a reduction in masticatory operation. To resolve these concerns and enhance patient contentment with their prostheses, an implant-supported overdenture was introduced [2].

Mandibular implant-retained overdentures can be an appropriate therapy for edentulous individuals, especially for those who have trouble using a traditional mandibular denture on a regular basis. Overdentures supported by implants provide greater stability, support, and retention of mandibular dentures. Additionally, it appears to be recommended for individuals who cannot afford permanent prostheses, as this option necessitates fewer implants, involves a less complex surgical procedure, and allows for the utilization of prefabricated attachments, thereby facilitating a more straightforward restorative method [3].

Clinicians have selected different attachment systems based on factors such as durability, patient demand, cost effectiveness, technical simplicity, and retention. Attachments can be classified depending on their function as rigid, if they do not allow any denture dislodgements, or resilient, when they allow translation, rotation, axial or hinge over posterior axe movements, or a combination of them because of their flexibility [4].

Because they are simple to use, require minimal chairside time, and may be used in a variety of settings without requiring the fabrication of new dentures, ball and socket attachments are among the most often used attachments presently [5]. It has also been demonstrated that, of the stud attachments, the ball attachment transfers the least pressures and may promote the health of the surrounding bone [6].

Because of its special qualities, such as low density, aesthetics, affordability, ease of manipulation, and adaptable mechanical and physical properties, polymethyl methacrylate (PMMA) is a preferred biomaterial for many dental applications. As a result, PMMA is a popular and appropriate biomaterial for complete denture construction [7]. PMMA still has issues with poor surface characteristics despite its strong performance. Microorganisms can stick to the surface, proliferate, develop biofilms, and enter the denture through pores, fissures, and structural flaws caused by the release of monomers during the polymerization process. This is particularly common at the contact between the denture and the attachment [8].

Dental implant-related diseases such as peri-implantitis can be brought on by microbial adhesion and attachment. Bone resorption and implant loss are potential consequences of peri-implantitis [9]. There have been attempts to alter PMMA in an effort to enhance the mechanism underlying its antibacterial properties. Fiber materials, fillers, and metallic nanoparticles are some of these alterations [10].

Gold nanoparticles (AuNPs) are one type of metallic nanoparticle that has garnered more attention than other types because of its advantageous properties. Low toxicity, compact size, accurate concentration, and rather easy manufacture are some of these qualities. Various approaches have been employed to demonstrate the antimicrobial characteristics of AuNPs [11].

The utilization of gold nanoparticles (AuNPs) in implant components, dental devices, prosthetic structures, and orthodontic aligners may significantly improve the biological response as well as the antibacterial characteristics. The antimicrobial properties of nanoparticles can be attributed to their extremely small size, which can be up to 250 times smaller than that of bacteria. The small size of AuNPs can lead to electrostatic interactions between the Au atom and the negatively charged cell wall of the microbes. It was showed that incorporation of Au NPs into different polymeric materials reduced the growth of E. coli by a factor of 106 compared to unmodified surfaces. Several authors also noted a pronounced antifungal property of Au NPs on different Candida species, such as C. albicans, C. glabrata, and C. tropicalis [12, 13].

In light of this, the goal of this research was to investigate the effect of mixing PMMA with AuNPs to produce a novel substance that would inhibit the formation of microbial biofilms and shield the oral tissues from harmful bacteria and yeasts. Comparing the microbiological adhesion and colonization beneath mandibular implant retained over denture bases made from modified PMMA by adding AuNPs to the non-modified conventional PMMA.

## Methods

### Study design and sample size calculation

This randomized clinical trial was two groups, single-blinded, parallel design study. Based on Maricʹ (2023) [14], using G power statistical analysis program (Version 3.1.9.4) for sample size calculation and using difference between two independent means (two groups), A total sample size *N* = 22 (subdivided into 11 in each group) was sufficient to detect a large effect size = 1.29 with an actual power (1-β error) of 0.8 (80%) and primary risk of error (α = 0.05).

### Patients’ selection criteria

The patients chosen were from the removable prosthodontic department’s outpatient clinic, faculty of dental medicine for girls, Al-Azhar University. They were between the ages of 50 and 60, had firm mucosa covering their remaining ridge; there were no signs of irritation or ulceration. Their salivary flow and viscosity were all within normal limits, and they were cooperative and willing to be recalled as needed. Patients who were smokers or who had used antifungal, antibiotic, or antidepressant medications within the six months before the start of the study were not allowed to participate.

### Patients grouping

Using a balanced random method, the patients were randomly assigned into two groups, with eleven patients in each group, to guarantee that there weren’t any differences in patient characteristics at the start of the study. Eleven participants in Group I (conventional dentures) got mandibular overdentures with conventional acrylic denture bases. Eleven participants in Group II (AuNPs dentures) received mandibular overdentures made of nanogold-treated denture bases. Each patient received an explanation of every operation, and informed permission was obtained.

### Ethical consideration

The proposal and consents were approved by the research ethics committee (REC), faculty of dental medicine for Girlz, Al Azhar university. (REC) approval (REC-PD-24-09).

### Clinical and radiographic examination

Radiographic and clinical assessments were performed. For each patient, a radiographic template was constructed. The evaluation of buccolingual width and bone height, essential for implant placement, was conducted through the use of a cone beam radiograph (cone beam machine model Planmeca Paromax 3D, Finland). The bone height ‘s mean and standard deviation (SD) for the study subjects was 16.4 + 2.1 mm.

### Complete dentures construction

Preliminary impressions were obtained utilizing an irreversible hydrocolloid impression material (Cavex CA37, Holland). Following the border molding process with Putty-C-Silicone, a silicone impression material (poly-C-silicone impression material, thixoflex M, medium, Zhermack, Italy) was employed to produce the final impression. Once cleaned, the impressions were placed in a container and subsequently filled with dental stone (Dental Stone is a Spanish company that produces hard stones). The maxillary cast was mounted on the articulator by using the maxillary face bow (Hanau model H articulator and face bow, Teledyne Dental, Hanau Division Buffalo, NY, USA), and the mandibular cast was mounted using the patient’s centric jaw relation record, which was obtained at the prescribed vertical dimension of the maxillary cast. After positioning the artificial teeth that made of cross-linked acrylic resin (Acrostone Manufacturing Import, Egypt) in a balanced occlusion, the denture was waxed and inspected within the patient’s mouth.

## Denture processing

### For group I (conventional PPMA dentures)

The maxillary and mandibular dentures were made by using conventional heat-cured acrylic resin (Acrostone Manufacturing, Egypt, licensed by WHW, England) in accordance with standard processing procedures [15]. Conventional procedures for packaging, curing, deflasking, finishing, and polishing were also followed.

### For group II (AuNPs dentures)

The composite material consisting of PMMA and gold nanoparticles sized 20 nm (AuNPs, Nano Composix, San Diego, ) was synthesized by integrating liquid AuNPs into commercially available PMMA (Acrostone manufacture, Egypt, Licensed by WHW, England). The technique of making PMMA and AuNPs composite was followed by previous study [16]. The AuNPs dissolved in ethanol at a concentration of 1 g/L. For every 23.4 g of PMMA powder, a total of 15 ml of liquid mixture was added, which included 10 ml of PMMA monomer and 5 ml of AuNPs. Polyvinilyrolidone (PVP) was employed to stabilize the solution. The preparation of both components adhered to the manufacturer’s guidelines: the powder and liquid were blended until a uniform dough was achieved, which was then packed into molds. After that it was sealed under a pressure of 80 bars and immersed in water, which was gradually heated to 100 °C. After maintaining this temperature for 1 h and 45 min, the system was allowed to cool gradually to room temperature, and then dentures were deflasked, finished, and polished in accordance with the standard protocol.

## Surgical procedures

### First stage surgery

The surgery was done by employing a standardized two-phase submerged surgical approach as follows: the patient received a mouthwash containing chlorhexidine gluconate two days prior to the surgical procedure. Also, Amoxicillin Clavulanate Potassium was prescribed at a dosage of 2 g per day, commencing the day before surgery and continuing through the fifth postoperative day. Subsequently, the metal balls were extracted, transforming the radiographic template into a surgical stent. Under local anesthesia, dental probes were utilized to establish bleeding points on both sides of the jaw, while the surgical stent was carefully positioned on the ridge to ensure accurate alignment of the osteotomy sites. A crestal incision, along with two vertical incisions in the canine area, was performed using scalpel no. 15, extending deep to the bone. Following this, a full-thickness mucoperiosteal flap was reflected with the aid of a mucoperiosteal elevator.

### Implant installation

The implant’s type, width, and length were standardized for each patient. Two endosteal implants that taper and self-tape (TCR Multisystem, Italy) measuring 11.5 mm in length and 3.2 mm in diameter have been inserted bilaterally in the canine region of the mandible for each patient. After the surgical stent was inserted, the location of the entrance was determined using a locating drill. Two pilot drills were utilized at the predefined implant position. (Multisystem implant kit, Italy) measuring 1.8 and 2.3 mm in diameter. The pilot drill was only used to make a hole that was around one-third of the required length during this first drilling phase. After that, the alignment pin was inserted to verify the orientation of the hole that had been made, and it was continued at speed 800 to fit the entire intended implant. The site was expanded using a final drill bit (2.8 mm) to reach the desired depth and width.

Following the preparation of the implant site, the selected implant was subsequently inserted. Next, each implant was carefully screwed in a clockwise manner using a ratchet (Multisystem implant kit, Italy). After the level of the crestal bone had been achieved, the installation was maintained until resistance was encountered. After that, the implants’ cover screws were tightened. The second flap was completed in a similar fashion, utilizing the surgical stent as a reference. The flap was accurately realigned and secured with black silk sutures. Additionally, the initial fixation proceeded without complications in all participants involved in the study.

After surgery, the patient was instructed to take non-steroidal anti-inflammatory medications to reduce pain and infection risk, to follow a five-day antibiotic medication (one gramme amoxicillin every twelve hours), to rinse twice a day with chlorohexidine mouthwash to prevent the plaque accumulation, and to follow a soft diet for the first week.

### Second stage of the surgery

The second part of the surgery was carried out under local anesthesia three months later. The surgical template was used to pinpoint the implant’s location. A healing abutment that had an appropriate height determined by the soft tissue thickness was placed and manually screwed down with a screwdriver after a tissue punch (Multisystem implant kit, Italy) was made over the implant fixture and the covering screws were removed. The healing abutment was left in place for seven days to permit the healing of the gingival tissue surrounding the implant.

### Attachment installation

**Fig. 1:**
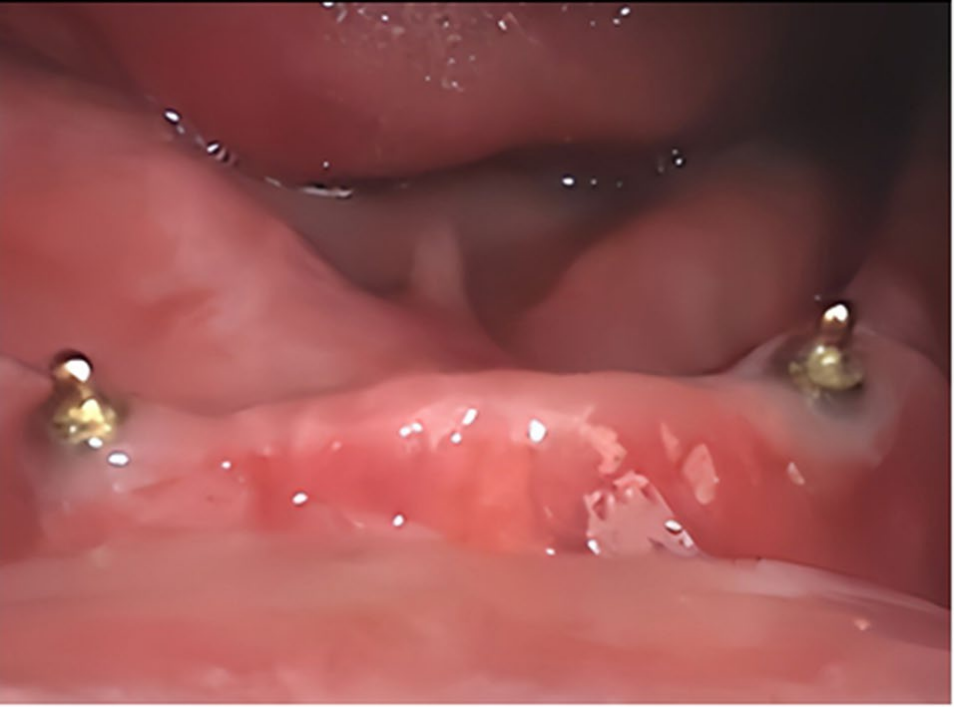
Attachment installation over the mandibular implants

The standardized ball and socket attachments were utilized for each patient in the following manner: The male ball abutment (Multisystem, Italy) was equipped with the supplied metal housing, which consisted of the male component, medium (M), with a gingival height of 3 mm and a ball diameter of 2.25 mm. The outer matrix (the female components, Matrix Unor Ecco Au/Pt standard) was placed and fastened to the implant fixtures using an abutment driver Fig. 1.

### Denture pickup

**Fig. 2:**
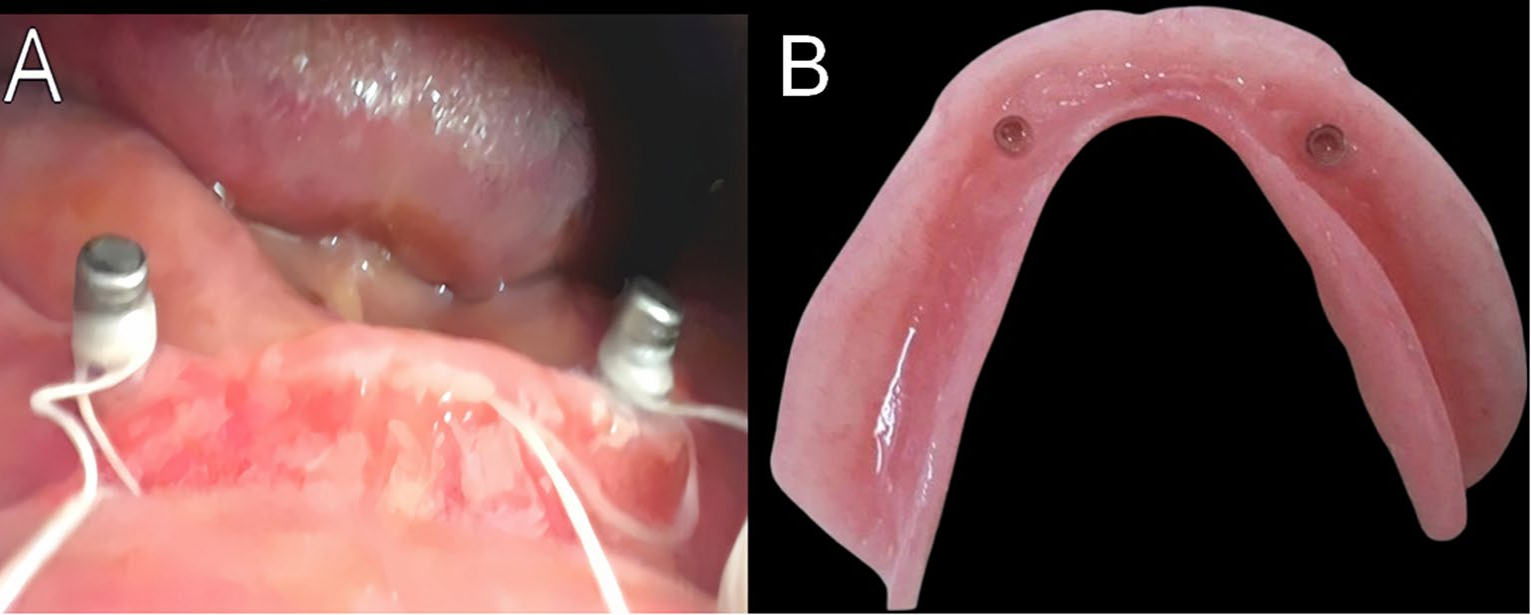
**(A)** Dental floss was used to block the undercut. **(B)** The socket attachment was picked up into the denture fitting surface

The socket attachments were picked up intraorally as follows: dental floss was used to block the undercut, which serves as a barrier to stop extra acrylic resin from entering the sulcus deeply during the pickup process. Figure 2A The denture was appropriately relived in the opposite direction of the attachment point, and its correct occlusion and lack of rocking were assessed. Following the manufacturer’s instructions, cold-cured resin (Acrostone manufacture, Egypt, licensed by WHW, England) was prepared, applied to the denture’s attachment regions, The acrylic resin was allowed to polymerize while the patient was closing in the centric jaw relation. Then the socket attachment was picked up into the denture fitting surface, the excess acrylic resin was removed, and the denture was finished.

Following an examination of denture for retention, stability, extension, articulating, and simplicity of insertion and removal, the patient received the denture. The patient received home denture maintenance instructions as well as guidance on maintaining good oral hygiene Fig. 2B.

### Microbial assessment

Samples were taken from the area around the neck of implants and the fitting surface of each patient’s mandibular dentures that was opposite to the implant by using disposable gamma-sterilized swabs. The samples were collected before the denture was inserted, two, four, and six months later. The patients were told not to remove their dentures four hours before the sampling and not to alter their regular oral care routines.

Sterile tubes were filled with sterile saline. Five tubes were used for every sample. One tube held 1000µL of sterile saline, while the remaining four contained 900µL. Following an initial 15 min of incubation in the first sterilized tube with 1000µL of sterile saline, each swab had been diluted at 1/10, 1/100, 1/1000, and 1/10,000. The selective agars used for microbial cultivation included blood agar base No. 2, which is appropriate for the growth of germs and other microbes; MacConkys agar, which is a differential medium for the isolation of gram-negative microorganisms and Sabouraud’s dextrose agar, which is selective for the isolation of fungi. For every tube, a 100-µL sample was placed on the proper agar substrate.

The anaerobic bacteria were grown in anaerobic jars, and all the microbes were cultivated for 24 h at 37 °C. The bacteria that are being investigated include Candida albicans (C. albicans), Escherichia coli (E. coli), and Streptococcus mutans (S. mutans). The number of clusters on the agar gels’ outer layer was counted to verify the microbial evaluation, and log 10 CFU/mL was used to compute the colonies forming units per milliliter (CFU/mL).

For groups I and II, the counts of bacteria after two, four, and six months has been calculated, tabulated, and analyzed statistically.

## Results

### Statistical analysis

The SPSS (Version 26) software package was utilized for statistical analysis. The data were presented as mean and standard deviation (SD) values. A logarithmic transformation (log 10 transforming) of each colony-forming unit (CFU) count was performed to normalize the data before statistical evaluation because of the high range of counts. Differences in CFU numbers were analyzed using repeated measures ANOVA for intragroup comparison and an independent sample t-test for intergroup comparison. The significance level was set at *P* ≤ 0.05.

I. **Comparison within each group regarding bacterial colony count (log 10 CFUs / mL) during follow up period:**

Table 1 represents bacterial colony count at 2 months, 4 months and 6 months, respectively (log 10 CFUs/mL) in both groups.

#### For group I

The measured values varied significantly from one another at 2 months, 4 months, and 6 months, respectively, for Candida albicans, E. coli, and S. mutans. (*P* < 0.001) The highest values of colony number were recorded at 6 months for three bacterial types. Post hoc pairwise comparisons showed values measured at 6 months periods to be significantly different from 2 months and 4 months periods.

#### For group II

The measured values varied significantly from one another at 2 months, 4 months, and 6 months (*P* < 0.001), (*P* = 0.001), and (*P* = 0.017) for Candida albicans, E. coli, and S. mutans, respectively. The highest values of colony number were recorded at 6 months for three bacterial types. Post hoc pairwise comparisons showed values measured at 6 months periods to be significantly different from 2 months and 4 months periods.

**Table 1:** Bacterial colony count (log 10 CFUs / mL) at 2, 4 and 6 months, respectively in both groups

Bacteria	Group I (mean ± SD)				Group II (mean ± SD)			
	2months	4 months	6months	*P* value	2months	4months	6months	*P* value
Candida. Albicans	3.91 ± 1.6^A^	4.2 ± 0.99^A^	6.8 ± 1.17^B^	*P* < 0.001*	2.26 ± 0.50^A^	3.20 ± 0.95^A^	4.73 ± 0.64^B^	*P* < 0.001*
E. coli	4.63 ± 1.35^A^	5.93 ± 0.92^A^	7.63 ± 0.96^B^	*P* < 0.001*	2.62 ± 0.85^A^	3.33 ± 0.53^A^	4.43 ± 0.49^B^	*P* = 0.001*
S. mutans	4.43 ± 1.12^A^	5.93 ± 0.81^B^	7.03 ± 0.46^C^	*P* < 0.001*	2.73 ± 0.81^A^	3.33 ± 1.05^A^	3.83 ± 0.74^B^	*P* = 0.017

Within the same horizontal row, different superscript letters denote statistically significant differences. *; significant (*p* ≤ 0.05) ns; non-significant (*p* > 0.05)

II. **Comparison between two groups regarding bacterial colony count (log 10 CFUs / mL):**

**Fig. 3:**
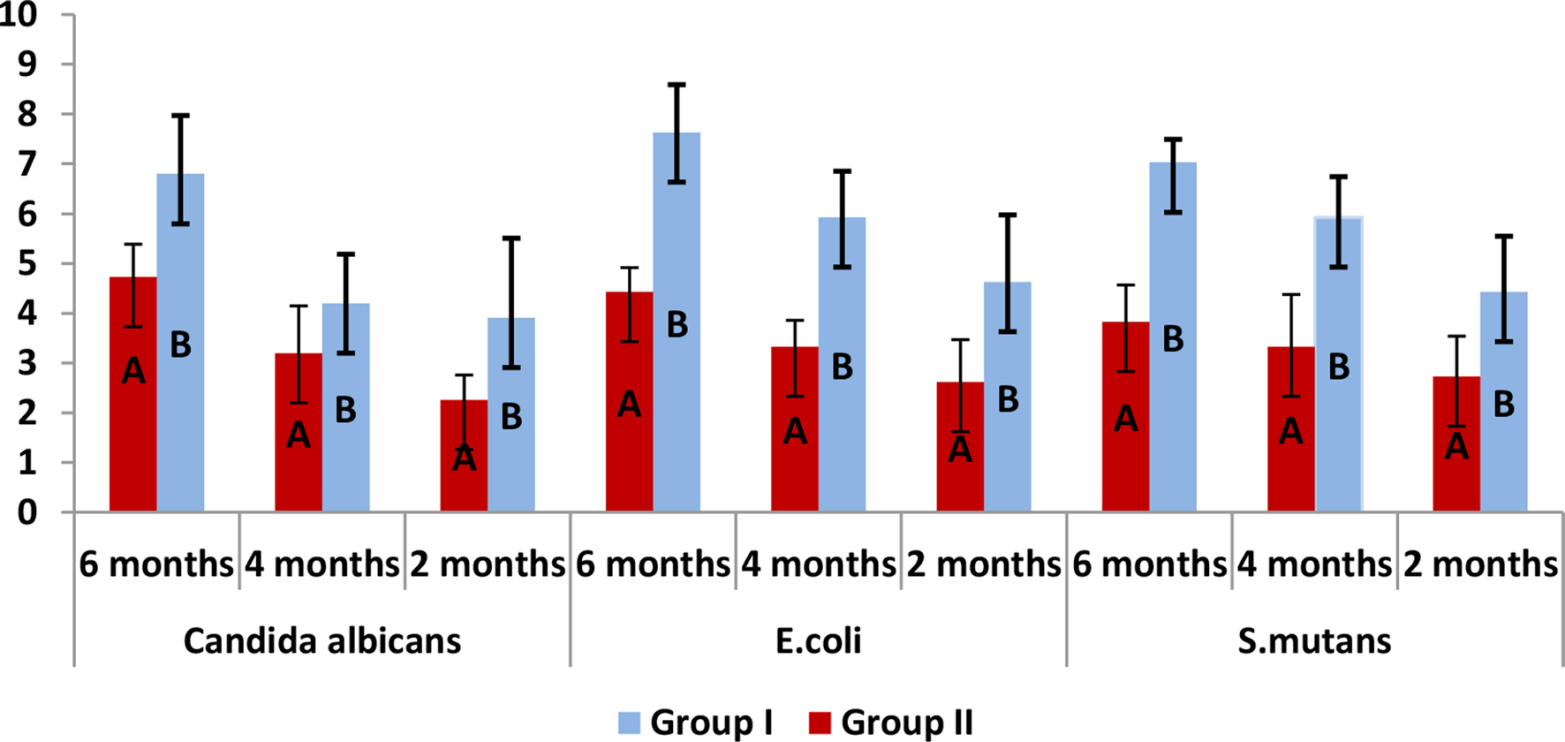
Represents intergroup comparison of bacterial colony count (log 10 CFUs/mL) in two groups

Table 2; Fig. 3: represents intergroup comparison of bacterial colony count (log 10 CFUs/mL) in two groups. For both groups: Group II had significantly lower values measured at 2 months, 4 months and 6 months for Candida albicans, E coli and S mutans respectively than group I (*P* < 0.001).

**Table 2:** Represents intergroup comparison of bacterial colony count (log 10 CFUs/mL) in two groups

Bacteria	Interval	Bacterial Colony count (log 10 CFUs / mL) (mean ± SD)		P-value
		Group I	Group II	
Candida albicans	2 months	3.91 ± 1.6	2.26 ± 0.50	*P* < 0.001*
	4 months	4.2 ± 0.99	3.20 ± 0.95	*P* < 0.001*
	6 months	6.8 ± 1.17	4.73 ± 0.64	*P* < 0.001*
E. Coli	2 months	4.63 ± 1.35	2.62 ± 0.85	*P* < 0.001*
	4 months	5.93 ± 0.92	3.33 ± 0.53	*P* < 0.001*
	6 months	7.63 ± 0.96	4.43 ± 0.49	*P* < 0.001*
S. mutans	2 months	4.43 ± 1.12	2.73 ± 0.81	*P* < 0.001*
	4 months	5.93 ± 0.81	3.33 ± 1.05	*P* < 0.001*
	6 months	7.03 ± 0.46	3.83 ± 0.74	*P* < 0.001*

*; significant (*p value* ≤ 0.05) ns; non-significant (p value > 0.05)

## Discussion

Despite few in number, in vivo studies are useful in examining the microbial activity in implant-retained denture bases so the purpose of the research was to examine the effects of adding gold nanoparticles to PMMA acrylic resin denture bases on peri-implant microbial colonization.

AuNPs are chosen due to their advantageous properties, such as stability, non-toxicity, uniform particle size, and antibacterial features, gold nanoparticles (AuNps) represent a highly suitable choice for use as fillers in nanocomposites. Also, it was indicated that gold nanoparticles, due to their distinct advantages compared to other nanoparticle types, exhibit significant biocompatibility and minimal cytotoxicity [17, 18].

There is a concentration limitation for nanoparticles in order to show their best antimicrobial properties [19]. The size of AuNPs used in this study was 20 nm. It has been shown that small particles smaller than 5 nm (especially 1–1.5 nm in size) have been reported to be highly toxic. The toxicity of those larger than 5 nm in size depends on the surface coating, synthesis method, gold concentration (µg Au/ml), and exposure time [20]. In previous studies using gold nanoparticles of different sizes (2–25 nm) without any additional surface functionalization, it has been shown that nanomaterials are mostly biocompatible [17, 21].

The bacteria that are the subject of the present study were selected because Candida albicans is thought to be the primary pathogen responsible for denture stomatitis, affecting roughly 67% of denture wearers [22, 23]. Additionally, the resin can be penetrated by C. albicans, creating a microbial reservoir [24]. Also, E. coli is considered a member of the transitory microbiota; it can encourage yeasts’ early adherence to the denture’s surface [25].

The last one is S. mutans which plays an important role in establishing an acidic media other acidogenic and aciduric microbes, which in turn leads to the emergence of complicated pathogenic biofilms. Also, S. mutans is systemically associated with serious medical problems including bacterial endocarditis and atherosclerosis [26], so evaluating S. mutans’s adherence to denture surface is clinically significant because of its various metabolic implications.

Additionally, it was indicated that Streptococci bacteria serve as the primary colonizers due to their capacity to adhere to both tooth surfaces and various solid structures within the oral cavity. Among these, S. mutans is particularly significant during the initial phases of colonization, facilitating the attachment and subsequent colonization of more pathogenic bacteria within the established biofilm. In contrast to natural teeth, dental implants do not possess inherent niches for bacterial biofilm formation, thereby elevating the importance of early colonizers like S. mutans in initiating biofilm development on the implant surface, which can trigger inflammation and ultimately result in peri-implantitis [27, 28].

Peri-implantitis is a pathological condition that impacts the tissues surrounding dental implants. It is characterized by inflammation and a progressive loss of supporting bone, which is attributed to an imbalance in the bacterial ecosystem. This imbalance is influenced by various factors that require extensive and prolonged investigation to yield accurate results in future research [29].

The specimen extracted from the region around the neck of implant and from the fitting surface of denture base as the subgingival region of the implant neck is one of the small, inaccessible locations that patients cannot reach despite following stringent dental hygiene habits, and this promote to the formation of bacterial invasion [30]. In addition, it has been highlighted that the bacterial species which present at the peri- implant level can then colonize and adhere to the prosthetic artifact [31].

As the study denotes an increase in microbial colony during the follow-up period in groups I, this coincides with many studies (32–33) justifying this with surface roughness (porosity), which is one of the material properties that influences the formation of biofilms and bacterial/fungal adherence. Surface porosity improves the possibility of microbe retention and also serves as a micro biofilm reservoir. Surface roughness can be influenced by various causes, including residual monomer, which vaporizes and increases surface roughness [34].

Similarly, the result showed that there was an increase in microbial colony during the follow-up period in Group II, which may be related to the permissible dispersion of nanoparticles inside the resin matrix. In addition to their capacity to occupy the spaces between polymer chains, there were fewer nanoparticles on the specimen’s surface [35].

Moreover, it was reported that there had been a significant decrease in bacterial colonization with the use of AuNPs. This is due to the smoother surface of PMMA/Au compared to PMMA, which reduces the adhesion of bacteria, as mentioned by Ivanovic V et al. [16], who prove that the addition of Au NPs improves the base material’s resistance to C. albicans adhesion by 66% after 24 h and 43% after 48 h. Also, evidence suggests that gold nanorods possess significant antifungal activity against yeast and filamentous fungi, with effective concentrations varying from 0.039 to 1.25 µg /ml [36].

It was demonstrated that gold nanoparticles possess considerable antibacterial properties against Gram-negative bacteria, including E. coli. Once these nanoparticles breach the bacterial cell wall and infiltrate the cytoplasm, they make direct contact with the cell membrane, especially in instances where the cell wall is compromised and the gold nanoparticles at concentrations of 0.1 µg/mL and 1.5 µg/mL demonstrated an efficacy in eliminating E. coli, achieving a remarkable effectiveness rate of up to 90%. Furthermore, an additional proposed mechanism suggests that these nanoparticles can modify pH levels; they promote acidification of the bacterial surroundings, resulting in a toxic pH that adversely affects bacterial viability [37, 38].

Morphological and nanomechanical changes to the C. albicans cells that based on linking of AuNPs to cell membrane that cause the alternation and structure deficiency and the function of cell like penetrating feature, and also by producing cracks and holes influencing on enzymes of respiratory chain causes cell death [39].

For S.mutans it was thought that nanoparticles can attach to the cell membrane and disturb the permeability of the outer membrane. Therefore, they can enter the inner layer of membrane and stop respiratory chain dehydrogenase, disassociate the respiratory chain and oxidative phosphorylation and disable protonmotive force via cytoplasmic membrane ^(18)^. It was observed that AuNPs exhibited an inhibitory effect on S. mutans at a minimum concentration of 1.73 ± 1.23 µg/ml [40].

The results are in accordance with some studies [41, 42] that describe the reduction of the bacterial biofilm of PMMA/Au to the antibacterial impact of Au NPs, as AuNPs’ antibacterial properties have been established in a variety of ways.

## Conclusion

It could be justified to draw the conclusion from this study that, in mandibular implant-retained overdentures, the addition of gold nanoparticles to PMMA denture bases was more beneficial in preventing microbial development and colonization than normal acrylic resin bases over the follow-up period.

## Limitations and recommendations

The findings of this research may be influenced by various factors, such as the duration of denture use and the practices of oral hygiene. These elements should be considered during the follow-up period, as they significantly affect bacterial adherence and colonization. This study is limited to evaluating the influence of nano gold addition to a conventional heat-polymerized PMMA. Consequently, additional research is necessary to investigate different formulations of the material and their interactions with other microorganisms, ideally over longer observation periods.

## Data Availability

The datasets used and analyzed during the current study are available from the corresponding authors on reasonable request.

## Abbreviations

PMMA: Polymethyl methacrylate
AuNPs: Gold nanoparticles
CFUs: Bacterial Colony forming unites
REC: Research ethics committee
E. coli: Escherichia coli
S. mutans: Streptococcus mutans
